# Cyclic Fatigue of Different Ni-Ti Endodontic Rotary File Alloys: A Comprehensive Review

**DOI:** 10.3390/bioengineering11050499

**Published:** 2024-05-16

**Authors:** Dina Abdellatif, Alfredo Iandolo, Michela Scorziello, Giuseppe Sangiovanni, Massimo Pisano

**Affiliations:** Department of Medicine, Surgery and Dentistry “Scuola Medica Salernitana”, University of Salerno, 84081 Baronissi, Italyaiandolo@unisa.it (A.I.); scorziellomichela96@gmail.com (M.S.); gsangiovanni7@gmail.com (G.S.)

**Keywords:** Ni-Ti files, Ni-Ti rotary instruments, Ni-Ti endodontic fractures, Ni-Ti file cyclic fatigue

## Abstract

Introduction: Modern endodontics aims to decrease the bacterial load from the complex endodontic space. Over the years, improvements in the operative phases have led to a considerable increase in the success rate of endodontic treatments. The shaping phase has seen the development of new techniques supported by technological innovations that have led to higher treatment predictability. Endodontic instruments have experienced a series of changes that have led to modifications in their design, surface treatments, and heat treatments. The clinical use of rotating nickel–titanium instruments has become widespread and consolidated, a success due primarily to the alloy’s mechanical characteristics, which are superior to steel ones, but also to innovations in instrument design. The advent of the Ni-Ti alloy has kept the concepts and requirements of shaping the same but has modified its implementation in endodontics. Aim: The following review followed the Preferred Reporting Items for Systematic Reviews and Meta-analyses (PRISMA) protocol. The research question focused on Ni-Ti endodontic instruments whose cyclic fatigue was evaluated by analyzing cyclic fatigue strength and the incidence of fracture. Results: At the end of the research, 10 systematic reviews and 1 randomized controlled trial were included in this comprehensive review. The most frequently analysed alloys were M-wire, conventional Ni-Ti, and CM-wire. In seven articles, instruments made of M-wire alloy were used; in eight articles, instruments made of conventional Ni-Ti; and in seven articles, instruments made of CM-wire alloy. Conclusions: The technological evolution of Ni-Ti alloys has led to the development of increasingly high-performance endodontic files that are resistant to cyclic fatigue during clinical practice and have greater resistance to sterilisation practices, making treatment easier and more predictable over time. In particular, heat-treated nickel-titanium root canal instruments present greater resistance to cyclic fatigue than untreated ones and those used with reciprocating kinematics concerning continuous rotation.

## 1. Introduction

Modern endodontics aims to decrease the bacterial load from the complex endodontic space [1]. Endodontic treatment involves several steps to lower the bacterial load and eliminate pathological pulp tissue to achieve long-term clinical healing [2]. Furthermore, it is essential to perform a predictable and standardised protocol that includes an adequate access cavity, followed by a shaping phase for the mechanical removal of infected dentin [3]. This protocol is followed by the three-dimensional cleansing of the intracanal complex anatomy using activated irrigants [4]. The obturation phase is performed to form a proper apical seal to avoid the reinfection of the root canal area [5].

To date, the available modern technology, including the most contemporary protocols, allows us to achieve a considerable reduction in the intracanal bacterial quota without, however, sterilising the complex root canal system [6]. Over the years, endodontic shaping has seen the development of new approaches supported by technological innovations that have led to a higher predictability of treatment [2,7]. Generally, the mechanical phase consists of several stages, including canal probing; pre-flaring, which is an initial coronal widening; canal scouting; working length in which manual files and an apical locator are used to calculate the working length; glide path, which consists of creating an initial contour; and shaping in which the canal is shaped [8]. Adequate shaping is crucial to achieve optimal cleansing by allowing irrigants to reach the apical third as well as a correct three-dimensional filling [9]. On the other hand, excessive shaping could cause the weakening of the element with consequent susceptibility to fracture [10].

In recent years, endodontic instruments have undergone a series of changes that have led to modifications in their design, surface treatments, and heat treatments [11]. 

The clinical use of rotating nickel–titanium (Ni-Ti) instruments has become widespread and well established. This success is primarily attributed to the mechanical characteristics of the alloy, which are superior to those offered by steel, and the innovations in instrument design. These instruments have significantly enhanced the efficiency and effectiveness of endodontic procedures, underscoring their importance in modern endodontics [12]. 

The advent of Ni-Ti alloy has kept the concepts and requirements of shaping the same, but it has modified its implementation in endodontics [7]. In dentistry, Andreasen and Hilleman [13] first used Ni-Ti instruments in orthodontics in 1971 due to their low modulus of elasticity, shape memory, and flexibility. In endodontics, Civjan et al. introduced the first Ni-Ti instruments in 1975, and currently, more than 160 instrument systems are made from different Ni-Ti alloys [11].

According to Hooke’s law, most metal alloys can be elastically deformed up to 0.1 or 0.2% beyond their elastic limit; any deformation beyond this limit, known as the yield point, becomes permanent. However, some types of Ni-Ti alloys exhibit a unique property called “hyperelasticity”. This means they can be elastically deformed up to 8% without experiencing residual deformation over a limited temperature range slightly above the forming temperature [11].

By taking advantage of the superelasticity of these instruments, which allows them to return to their original shape even after significant deformation, it has been possible to have sections and coils with complex morphologies and to introduce the concept of increased taper. This means we can have instruments of a larger size, capable of maintaining the characteristics of flexibility and resistance suitable for the rotary instrumentation of curved canals, thus having a superior cutting capacity and excellent flexibility and resistance [14]. Added to this is improving shaping quality, mainly understood as reducing iatrogenic errors that are common when using stiffer steel instruments [15].

Increasing the taper of Ni-Ti instruments makes achieving adequate preparation cross-sectional diameters possible, thus improving the instrument’s ability to mechanically remove contaminants, and increasing the area over which irrigating solutions can exert their chemical action [16].

According to Thompson, the special properties of Ni-Ti alloys are due to a solid-state phase change between the austenitic and martensitic structures. The austenitic structure is particularly stable and is the phase in which the alloy is typically used. The martensitic structure, on the other hand, is more ductile but more unstable and is the phase that allows the alloy to exhibit its unique properties [17].

In fact, there are three phases in the Ni-Ti alloy: the austenitic cubic lattice phase, which is particularly stable and is the phase in which the alloy is typically used; the martensitic phase, which is more ductile but more unstable and is the phase that allows the alloy to exhibit its unique properties; and the intermediate phase, which represents the transitional phase between the previous two [18].

In their resting state, first-generation Ni-Ti instruments are generally in the austenitic and intermediate phases. However, they change their structure when subjected to mechanical stress, such as rotation within a channel. Initially, the R phase is changed to one of its intermediate forms. Then, a phase transformation occurs with the formation of martensite, which is the most elastic form but also the weakest and can, therefore, fracture under loads of a lesser magnitude [12]. For the transformation to occur correctly, the stress must be constant, and therefore, a handpiece that rotates the instruments with a suitable speed and torque is needed [19]. 

Over the years, given the need to control torque and speed, more attention has been paid to cutting capacity. This refers to the ability of the instrument to remove dentin efficiently without causing excessive stress or strain on the tooth structure. Second-generation instruments with negative blades and cutting angles or positive blades and cutting angles and increased instrument cores [20] have been proposed to enhance this cutting capacity. 

Subsequently, third-generation instruments have been proposed to optimise cutting efficiency, resulting in less dentinal sludge formation. This refers to the accumulation of debris and irrigants in the root canal during shaping, which can hinder the cleaning and disinfection process. These instruments also have less tendency to fracture due to loop enhancement [21].

Further development has occurred recently as new manufacturers have entered the market, offering new designs and innovative production processes, such as Hyflex EDM, or newly treated alloys, such as FireWire (fourth- and fifth-generation instruments).

The main objective of these treatments is to have a higher martensitic phase in the files at body temperature to obtain the maximum advantage of flexibility [22]. These heat-treated tools can also improve fatigue strength, make it easily deformed, and exhibit shape memory [23].

Therefore, this review aims to evaluate the cyclic fatigue strength of different types of Ni-Ti files. This is a crucial property as it determines the ability of the instrument to withstand repeated use without fracturing, which is particularly important in the demanding environment of the root canal system. We will analyse the data reported in the literature to provide comprehensive knowledge of this aspect of Ni-Ti files.

## 2. Materials and Methods

### 2.1. Study Protocol

The following review followed the Preferred Reporting Items for Systematic Reviews and Meta-analyses (PRISMA) (https://www.prisma-statement.org/prisma-2020-statement) (accessed on 13 December 2023) protocol [24,25,26].

The research question focused on Ni-Ti endodontic instruments, whose cyclic fatigue was evaluated by analysing cyclic fatigue strength and the incidence of fracture.

### 2.2. Search Strategy

An electronic search was conducted of systematic reviews dealing with the evaluation of cyclic fatigue in Ni-Ti rotary instruments published through 20 February 2024. Several registries and databases were used: MEDLINE/Pubmed, PROSPERO, Scopus, Cochrane Library, and BioMed Central. The search was conducted by two independent reviewers (G.S.) and (M.S.), integrating the reported keywords with Boolean operators (“Ni-Ti instruments” OR “Ni-Ti files” OR “Ni-Ti rotary instruments” OR “Ni-Ti rotary instruments” OR “Ni-Ti endodontic instruments”) In addition, (“cyclic fatigue” OR “fracture” OR “fracture incidence” OR “strength”). For the search conducted on the MEDLINE/PubMed database, “Systematic Review”, “Meta-analysis”, and “Randomized Controlled Trial” filters were applied, and for the Scopus library, the “Review” filter was applied. No filter was applied to the BioMed Central database, as well as to the Cochrane Library and the PROSPERO registry. An additional temporal filter was applied to search for articles published in the last ten years to avoid obtaining results based on outdated evidence.

### 2.3. Study Selection and Eligibility Criteria

Before conducting the electronic search, inclusion and exclusion criteria were established to select studies for the analysis properly.

Inclusion criteria:

- Reviews with or without meta-analysis comparing the cyclic fatigue strength of different rotating Ni-Ti instruments, including in vivo and in vitro studies.
- Reviews that have been published within the past ten years.
- Reviews published in the English language only.
- Randomized controlled trial published within the past ten years.

Exclusion criteria:

- Reviews that did not aim to assess fatigue in rotating Ni-Ti instruments.
- Case reports.
- Reviews with a publication date before ten years ago.
- Reviews published in languages other than English.

Zotero (Version 6.0.27) reference management software was used to exclude duplicates. Next, the two reviewers (G.S.) and (M.S.) made a selection independently based on reading the reviews’ abstracts. The articles with abstracts deemed relevant to the research were selected, and the full text was analysed. They were then screened by the two reviewers independently, and in case of doubt or disagreement, a third reviewer (A.I.) was approached. A manual search was then conducted for any titles of articles in the bibliographies of the selected journals that might be relevant to this review. No maximum number of studies included in the systematic review was set.

### 2.4. Data Extraction and Collection

The two independent reviewers (G.S. and M.S.) extracted the data from the selected articles, and in case of disagreement, a third reviewer (A.I.) was approached.

The following criteria were recorded for each systematic review included:

- The first author of the journal, the year of publication, the type of journal, and funding.
- The number and design of studies included in each systematic review and sample size.
- Alloy.
- Kinematics.
- Diameter/Conicity.
- Conclusions.

### 2.5. Quality Assessment

The quality of the included systematic reviews was assessed using the AMSTAR 2 tool (https://amstar.ca) (accessed on 6 February 2024). The two independent reviewers (G.S. and M.S.) made their decision of “yes”, “partial yes”, or “no” using the 16 items in the AMSTAR 2 checklist. Disagreements were resolved by a third reviewer (A.I.). The information included in each review was gathered based on what was reported from the systematic reviews. The data obtained were used to complete the AMSTAR 2 online checklist. A final categorisation of each systematic review was then generated to classify it as being of “high”, “moderate”, “low”, or “critically low” quality.

## 3. Results

### 3.1. Study Selection 

**Figure 1:**
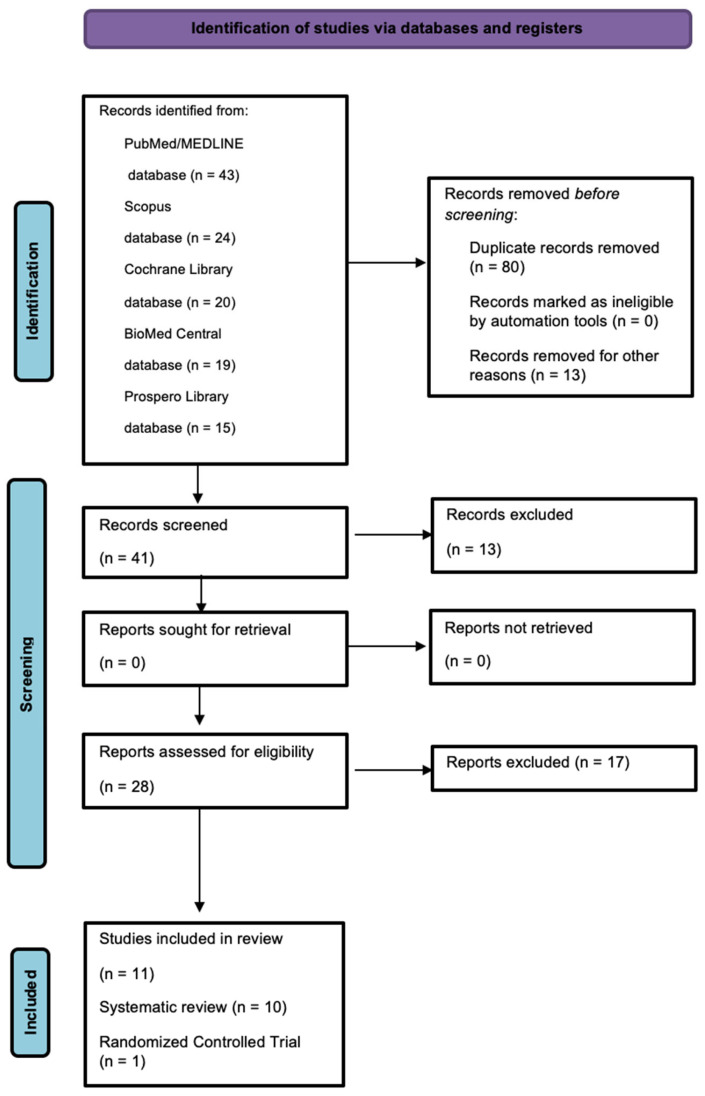
PRISMA flowchart of the screening process.

From the electronic search, 121 papers were found, of which 43 were from the MED-LINE/PubMed database, 20 were from the Cochrane Library database, 19 were on BioMed Central, 15 were on Prospero, and 24 were from Scopus. In total, after the elimination of 80 titles found to be duplicated, 41 abstract titles were examined. Of these 41 title abstracts, 13 title abstracts were excluded, and only 28 abstracts were relevant to this review. These full texts were examined, and 17 articles were found not to meet the inclusion criteria, so they were excluded. The study-selection flowchart is illustrated in Figure 1.

### 3.2. Study Characteristics and Qualitative Synthesis 

At the end of the research, the present review included ten systematic reviews and one Randomized controlled trial. Table 1 synthesises the characteristics and outcomes from included studies, Table 2 includes the total number of involved study participants and the total studies included in the reviews and Table 3 includes meta-analysis and related statistical data.

From the 121 articles analysed, ten were found suitable according to the inclusion criteria.

The most frequently analysed alloys were M-wire, conventional Ni-Ti, and CM-wire. M-wire alloy instruments were used in seven articles, conventional Ni-Ti instruments in eight articles, and CM-wire alloys in seven. 

In all articles, a continuous rotational movement was studied; in 10 articles, the reciprocating movement was also studied; and in 2, the adaptive movement. 

All articles consider in vitro studies, two in vivo studies, and only one ex vivo study. 

The total number of studies analysed from the reviews we included in the overview is 366. The total sample analysed from the selected articles turns out to be 6758. A total of 247 articles from in vitro studies with a sample size of 5967, 77 in vivo studies with a total sample size of 1075, and 58 ex vivo studies with a sample size of 1923 were included in the reviews.

## 4. Quality Assessment

The assessment using the Assessing the Methodological Quality of Systematic Reviews (AMSTAR) 2 tool found that most of the studies were to be judged as low to moderate quality. The ratings are shown in Table 4.

## 5. Discussion

Modern endodontics aims to reduce the bacterial load in the endodontic space as much as possible [1]. To accomplish this goal, all steps of root canal treatment must be executed correctly, from opening the pulp chamber to shaping, three-dimensional cleaning, and obturation. 

This review aims to analyse and summarise the data in the literature evaluating the cyclic fatigue strength of different Ni-Ti endodontic instruments with different kinematics. 

The current work considered three parameters:

- The cyclic fatigue resistance of heat-treated files.
- The fracture resistance of files used with different kinematics.
- The effects of sterilisation cycles on the degree of fracture of Ni-Ti instruments.

The large number of publications on this topic underscores the interest in developing increasingly high-performance files for more predictable endodontic treatment results. However, a significant portion of these published works are based on in vitro studies. It is crucial to underscore the need for more in vivo studies to support this evidence and help illuminate the limitations affecting the performance of Ni-Ti files. This call for further research not only highlights the urgency and importance of advancing our understanding in this field but also points to a potential area for future advancements.

### 5.1. Heat-Treated Files

Over time, the operational phases have improved with the evolution of rotary instruments, leading to increased success rates [2]. The instruments have been modified through surface treatments and heat treatments [11]. These modifications were necessary due to the risks of fracture associated with the introduction of the Ni-Ti alloy, despite its considerable advantages in mechanical properties. EDM treatments were introduced to address this issue [38].

Ni-Ti files are prone to fracture due to cyclic fatigue, which is not perceptible [39]. Heat treatments aim to influence the transition phases of Ni-Ti alloys, thereby enhancing the torsional and cyclic fatigue strength [40] to achieve a stable martensitic phase. The files are machined by creating a potential difference between the workpiece electrode and the tool electrode; the resulting sparks cause the surface material to melt and evaporate, leading to a more compact and fracture-resistant surface [41,42]. During production, austenitic and martensitic transitions occur at room temperature, enhancing flexibility, fracture resistance, and cutting capacity [43]. File fracture can occur through two mechanisms: torsional fatigue, which occurs when the tool rotates within the canal, and cyclic fatigue, when the metal is continuously exposed to compaction and tensile stress, leading to the deterioration of the file structure [44].

Selventhra et al. [29] analysed the effect of body temperature on the cyclic fatigue strength of Ni-Ti alloy instruments. Twenty-one studies were included, six of which underwent a meta-analysis with comparative study parameters analysing the distortion effect of heat-treated Ni-Ti files at room and body temperatures with continuous and reciprocating motion. The conclusions showed that the cyclic fatigue strength of heat-treated instruments decreases at body temperature compared to room temperature. Therefore, tests on these instruments should be performed at a temperature simulating the endodontic space [29].

### 5.2. Continuous Rotation vs. Reciprocating Motion

Rotating instruments can heat the irrigant and create turbulence with their movement. The Crown-Down technique contributes significantly to the rotary movement with its three steps: coronal access, the coronal and middle third, and then the preparation of the apical third [45]. 

The reciprocating movement is an evolution that arose to reduce the risk of cyclic fatigue fractures. It is based on the concept expressed by Roane, who introduced a movement for manual files to be performed during the shaping phase, characterised by a 270° clockwise movement and a 90° counterclockwise movement. This step is intended to increase the files’ cyclic fatigue resistance since the counterclockwise rotation of the movement decreases the torsional stress in root canal shaping [33]. 

Several studies evaluated the degree of fracture and cyclic fatigue strength of files by comparing continuous rotation with reciprocating motion. In all but three studies, both types of movement were analysed. Ashkar et al. [27] evaluated the difference between continuous rotation and reciprocating motion in different rotating instrument alloys. They included 25 studies that evaluated the difference in the cyclic fatigue strength of Ni-Ti files used with continuous and reciprocating rotation kinematics. Ashkar’s results showed significantly higher cyclic fatigue strength when using reciprocating kinematics while considering the factors influencing cyclic fatigue strength, such as file taper, cross-sectional design, channel curvature, irrigation, and temperature. They concluded that reciprocating motion results in higher cyclic fatigue strength, considering a higher number of cycles and time to fracture [27]. This evidence was also confirmed by Ahn et al. [33], who analysed 14 studies intending to assess not only the cyclic fatigue strength of Ni-Ti files used with different kinematics but also the degree of dentin debris extruded during the shaping phase. The results obtained showed a greater cyclic fatigue strength in Ni-Ti files used with reciprocating kinematics than those used with continuous rotation and, in addition, a greater tendency to extrude dentin and less tendency to form intracanal blocks during shaping with reciprocating kinematics [33]. 

Similarly, Ferreira et al. [28] also concluded that reciprocating motion improves cyclic fatigue strength. However, he adds that the need for more standardisation of experiments, as there are various reciprocating motions, prevents the creation of an unbiased scientific evidence base [28].

### 5.3. Effects of Sterilisation Cycles on Ni-Ti Files

In addition to heat treatment and the type of kinematics used, which influence the cyclic fatigue strength of Ni-Ti files, sterilisation steps, which are useful for killing bacteria on medical instruments (including endodontic files), were also analysed.

Autoclaving cycles lead to mixed results regarding the different effects of sterilisation procedures. In particular, shear capacity appears to change after five autoclaving cycles. The use of hypochlorite disinfectants will lead to a corrosive effect after the application of hot sterilisation. There is, however, a shape recovery effect after sterilisation. In any case, it also depends on the type of instrument. Dioguardi et al. [30], studying the effects of sterilisation, concluded that depending on the treatment, there is a different effect on the instruments as there is a reduction in shear capacity after autoclaving [30], which is reflected in a higher pressure by the endodontist with a higher probability of instrument fracture [35], a corrosive effect after use with sodium hypochlorite disinfectants. After sterilisation, there is a potential shape recovery effect and a higher resistance to torsional and cyclic fatigue [31]. M-wire and CM-wire exhibit improved reliability after sterilisation. 

Silva et al. [32] conducted a systematic review to evaluate the influence of autoclave sterilisation procedures on the cyclic fatigue strength of heat-treated Ni-Ti instruments. Five studies were included for the qualitative synthesis, and the results obtained confirm the change in post-sterilisation characteristics. It is also true that Silva et al. [32] emphasise the high risk of study bias and the low number of scientific and statistically significant trials.

### 5.4. Limitations of the Study 

The studies included in this review had different objectives and not all analysed and evaluated the same parameters. Results on the cyclic fatigue strength of heat-treated files, files used with different kinematics, and the behaviour of the files to sterilisation cycles are discussed. However, problems arise with reviews that analyse the same items. For example, reviews that analyse the fracture behaviour of files with different kinematics have different numbers of teeth or in vitro channels. A further limit is given by the non-standardisation and heterogeneity of the movements due to the different angles of use to which the files analysed in the different works included in the article were subjected. It should also be considered that the kinematics of endodontic motors can be set with different values compared to those declared by the manufacturers. The analysis using the Assessment of Methodological Quality of Systematic Reviews (AMSTAR) 2 tool [26] found that most of the studies were of low to moderate quality, and one was critically low quality from the assessment of methodological quality. It should also be noted that the included reviews have different settings and have a moderate to high risk of bias. This is also associated with a limited number of reviews evaluating in vivo versus in vitro studies, which do not faithfully reproduce the intraoral root canal environment. A further limitation is the lack of sample standardisation due to the inherent anatomical variables of the endodontic spaces of the teeth.

## 6. Conclusions

The predominant failure mechanism for Ni-Ti endodontic files was identified as cyclic fatigue caused by bending or torsion.

Based on the analysis of the systematic reviews included in this work, it was found that heat-treated Ni-Ti files are more reliable and less prone to fracture due to the accumulation of cyclic fatigue during the shaping phase, and this improves when associated with reciprocating kinematics as opposed to continuous rotation. 

The heat treatment and the type of kinematics used influence the cyclic fatigue strength of Ni-Ti files. It was found that the sterilisation phases also play a key role because while the cutting capacity of the files decreases after autoclaving, it is also true that, after sterilisation, there is a potential shape recovery effect and greater resistance to torsional and cyclic fatigue.

## Figures and Tables

**Table 1:** Key characteristics of included reviews: author, year of publication, reference, journal of publication, and type of review with meta-analysis (if any). Number and design of included studies, population, sample size, file type, and working kinematics; main outcomes and conclusions.

Author	Study/Purpose	Type of Study	No. Studies	Sample Size	League	Kinematics	Diameter/Taper	Conclusions
Israa Ashkar Materials (Basel) 26 September 2022 [27].	A review of in vitro studies. The aim of this systematic review was to provide a summary of in vitro studies analysing the cyclic fatigue strength of rotating files used for glide path.	Systematic review	20	1025	M-wire, Conventional Ni-Ti, Gold-wire, CM-wire, and Heat-treated firewire Ni-Ti	Continuous rotation and reciprocating motion.	10, 12, 13, 15, 16, 19, 20/2% 12/4% 12, 14, 17/3% 10/15%	The cyclic fatigue strength of files used for glide path can be influenced by intrinsic factors such as taper, cross-section, alloy properties, and kinematics, and external factors such as channel curvature, irrigation, lubricant use, and temperature. In addition, reciprocating motion results in a higher cyclic fatigue strength.
F. Ferreira Int. Endod. J. February 2017 [28].	A systematic review of in vitro studies. The aim is to analyse the correlation between different motion kinematics and the cyclic fatigue strength of Ni-Ti files.	Systematic review	32	1923	Conventional Ni-Ti, Gold Wire, and CM-wire	Continuous rotation and reciprocating motion.	19/4% 18/2% 20/4% 20/7% 25/8% 25/6% 30/6% 40/4%	The literature reviewed suggests that reciprocating motion improves the cyclic fatigue strength of Ni-Ti instruments regardless of the intrinsic and extrinsic file factors. However, the scientific evidence is not unbiased due to the lack of sample standardisation.
Selventhra S. J. Conserv. Dent. July–August 2022 [29].	A systematic review and meta-analysis of in vitro studies. The analysis of the effect of body temperature, compared to room temperature, on the cyclic fatigue strength of Ni-Ti instruments.	Systematic review	21	215	Heat-treated Ni-Ti and Conventional Ni-Ti	Continuous rotation, reciprocating motion, and adaptive.	20/0.04 25/0.04 30/0.04 35/0.04 40/0.04 20/04 25/0.08 25/0.06 30/0.05 30/0.01 25/0.08 25/0.06 25/0.07 25/0.08 10/0.02 25/0.6 40/0.06	At body temperature, the cyclic fatigue strength of heat-treated Ni-Ti files is significantly reduced compared to room temperature.
Dioguardi M. Materials (Basel). 8 July 2019 [30].	Systematic review with meta-analysis. The aim is to analyse whether and how disinfection and sterilisation procedures alter the cutting efficiency of endodontic instruments.	Systematic review	12		Conventional Ni-Ti, Nitiflex, Profile, R-face, and K-file	Continuous rotation.	30/02 25/06 25/04 35/04	New generation instruments such as those made of M wire, CM wire, and EDM alloys are more reliable after sterilisation than older generation instruments.
Dioguardi M. Front. Biosci. (Landmark Ed). 30 December 2021 [31]	A systematic review and network meta-analysis of in vitro and in vivo studies. In the following review, the effects of heat sterilisation on the torsional properties of steel and Ni-Ti alloy instruments are analysed.	Systematic review	51	368	Gold Wire, CM-wire, Conventional Ni-Ti, M-wire, and Heat-treated Ni-Ti	Continuous rotation.	10, 12, 13, 15, 16, 19, 20/2% 12/4% 12, 14, 17/3% 10/15%	The literature reports conflicting results on the effects of sterilisation procedures, which certainly produce changes in the physical and mechanical properties of instruments. In summary, the cutting capacity after five autoclaving cycles is significantly reduced. Hypochlorite disinfectants cause a corrosive effect after hot sterilisation. In some studies, a potential recovery of shape and increased resistance to torsional fatigue after sterilisation is contemplated.
Silva E.J.N.L. Restor. Dent. Endod. 31 March 2020 [32].	A systematic review of in vitro studies. The aim is to evaluate the effect of autoclave sterilisation procedures on the cyclic fatigue strength of heat-treated Ni-Ti instruments.	Systematic review	5	174	R-phase, CM-wire, M-wire, and Gold-wire	Continuous rotation.	25/0.06 20/0.06 20/0.04 25/0.04 40/0.04 25/0.08 30/0.06	Considering the limited scientific evidence and the considerable risk of bias, it is possible to conclude that autoclaving procedures appear to influence the cyclic fatigue strength of heat-treated Ni-Ti instruments.
Ahn S.Y.J. Endod. July 2016 [33]	A systematic review of in vitro and ex vivo studies. The purpose of this review is to make a comparison, in terms of cyclic fatigue strength, modelling capability, debris extrusion, cracks, and dentin defects, between reciprocating motion and continuous rotation.	Systematic review	58	1923	M-wire, Heat-treated, R-face, Ni-Ti conventional, and CM-wire	Continuous rotation, reciprocating motion, and adaptive motion.	25/8% 40/6% 50/5% 21/6% 40/8% 25/0.06 20/0.06 20/0.04 25/0.04 40/0.04 30/0.06	The reciprocating movement leads to better resistance to cyclic fatigue and less canal transport compared to instruments used with a continuous movement.
Alsilani R. J. Int. Soc. Prev. Community Dent. September–October 2016 [34]	A systematic review and meta-analysis of the literature of in vitro and in vivo studies. In this study, Reciproc and WaveOne are compared through a meta-analysis with different parameters.	Systematic review	26	707	M-wire, Heat-treated, and Wave one	Continuous rotation and reciprocating motion.	25/8% 40/6% 50/5% 21/6% 25/8% 40/8%	Reciproc has a higher cyclic fatigue than WaveOne. Further studies are needed, in particular randomised clinical trials comparing remodelling, clinical efficiency, and the possibility to reuse Reciproc and WaveOne with standardised samples.
Dioguardi M. Materials (Basel). 22 March 2021 [35].	Systematic review and network meta-analysis. The aim is to analyse changes in file cutting efficiency after disinfection and sterilisation procedures.	Systematic review	56	367	Flexofile, Ni-Ti conventional, CM-Wire, and M-wire	Continuous rotation and reciprocating motion.		Disinfection and sterilisation processes cause changes in the files, such as a reduction in cutting capacity after five cycles. Due to the heterogeneity of the measurement methods used, it is complex to perform a meta-analysis.
Herbst S.R. A Network Analysis. J. Endod. June 2019 [36].	A network analysis of in vitro studies. The objective of the study is to evaluate the networks of cyclic fatigue strength studies with the assumption that the intrinsic and extrinsic properties of the instruments guide the comparison.	Systematic review	85	/	Conventional Ni-Ti, M-wire, CM-wire, and Reciproc	Continuous rotation and reciprocating motion.		The comparator used leads to a moderate risk of bias. Factors such as sponsorship should be explored for more certain results.
Gambarini G. Clin. Ter. May–June 2018 [37]	First, to evaluate in vitro the performance of two different Ni-Ti rotary instruments in one molar case; then, to evaluate their resistance to cyclic fatigue, compared to new ones	Randomized controlled trial	/	25	ProTaper Next	Continuous rotation.	17/04 25/06	Since in previous studies ProTaper Next demonstrated a better resistance to cyclic fatigue than most nickel–titanium instruments, Horizen’s performance put them in a high rank amongst the most resistant nickel–titanium rotary instruments

**Table 2:** The total number of involved study participants included in the reviews and the total studies included in the reviews.

Author	The Total Number of Involved Study	Participants Clinical Studies Included
Ashkar I. [27]	1025	20
Ferreira F. [28]	1923	32
Selventhra S. [29].	215	21
Dioguardi M. [30]	/	12
Dioguardi M. [31]	368	51
Silva E.J.N.L. [32]	174	5
Ahn S.Y. [33]	1923	58
Alsilani R. [34]	707	26
Dioguardi M. [35]	367	56
Herbst S.R. [36]	/	85

**Table 3:** Meta-analyses included in the review.

Authors	Main Results	Statistical Analysis	*p*-Value
Selventhra S. [29].	The cyclic fatigue resistance of heat-treated Ni-Ti endodontic instruments is significantly reduced at body temperature compared with room temperature.	full rotary motion: [SMD]: 4.80; 95% CI: 3.04–6.56 reciprocating motion: [SMD]: 6.37; 95% CI: 3.63–9.11	*p*-value = 0.346
Dioguardi M. [31]	The cutting capacity of Ni-Ti instruments decreases after the 5 sterilisation cycles.	SMD value of 0.80 CI: [0.05, 1.55].	*p*-value = 0.04
Alsilani R. [34]	Reciprocs resist cyclic fatigue better than WaveOne	MD value of 0.95 CI: 5.25–13.14	*p*-value < 0.001

**Table 4:** The level of the evidence of the systematic reviews with meta-analysis is included according to the AMSTAR 2 tool.

Studies Selected	Question and Inclusion	Protocol	Study Design	Comprehensive Search	Study Selection	Data Extraction	Excluded Studies Justification	Included Study Details	Risk of Bias	Funding Sources	Statistical Methods	Risk of Bias in Meta-Analysis	Risk of Bias in Individual Studies	Explanation of Heterogeneity	Publication Bias	Conflict of Interest
Ashkar I. 2022 [27]	Yes	Yes	Yes	Yes	Yes	No	No	No	Yes	N/A	Yes	Yes	Yes	Yes	Yes	Yes
Ferreira F. 2017 [28]	Yes	Yes	Yes	Yes	Yes	No	No	No	Yes	N/A	Yes	Yes	Yes	Yes	Yes	Yes
Selventhra S. 2022 [29]	Yes	Yes	Yes	Yes	Yes	No	No	No	Yes	N/A	Yes	N/A	Yes	Yes	Yes	Yes
Dioguardi M. 2019 [30]	Yes	Yes	Yes	Yes	Yes	No	No	No	Yes	N/A	Yes	Yes	Yes	Yes	Yes	Yes
Dioguardi M. 2021 [31]	Yes	Yes	Yes	Yes	Yes	No	No	No	Yes	Yes	Yes	Yes	Yes	Yes	Yes	Yes
Silva E.J.N.L. 2020 [32]	Yes	Yes	Yes	Yes	Yes	No	No	No	Yes	Yes	Yes	Yes	Yes	Yes	Yes	Yes
Ahn S.Y. 2016 [33]	Yes	Yes	Yes	Yes	Yes	No	No	No	Yes	N/A	Yes	Yes	Yes	Yes	Yes	Yes
Alsilani R. 2016 [34]	Yes	Yes	Yes	Yes	Yes	No	No	No	Yes	N/A	Yes	Yes	Yes	Yes	Yes	Yes
Dioguardi M. 2021 [35]	Yes	Yes	Yes	Yes	Yes	No	No	No	Yes	N/A	Yes	Yes	Yes	Yes	Yes	Yes
Herbst S.R. 2019 [36]	Yes	Yes	Yes	Yes	Yes	No	No	No	Yes	N/A	Yes	Yes	Yes	Yes	Yes	Yes

## Data Availability

Data are contained within the article.
